# Bacterial community structure and function shift along a successional series of tidal flats in the Yellow River Delta

**DOI:** 10.1038/srep36550

**Published:** 2016-11-08

**Authors:** Xiaofei Lv, Bin Ma, Junbao Yu, Scott X. Chang, Jianming Xu, Yunzhao Li, Guangmei Wang, Guangxuan Han, Guan Bo, Xiaojing Chu

**Affiliations:** 1 Institute of Soil and Water Resources and Environmental Science, College of Environmental and Resource Sciences, Zhejiang Provincial Key Laboratory of Agricultural Resources and Environment, Zhejiang University, Hangzhou, 310058, China; 2 Key Laboratory of Coastal Environmental Processes and Ecological Remediation, Yantai Institute of Coastal Research, Chinese Academy of Sciences (CAS), Yantai, 264003, China; 3 Department of Renewable Resources, University of Alberta, Edmonton, Alberta, T6G2E3, Canada; 4 College of Resource and Environmental Engineering, Ludong University, Yantai, 264025, China.

## Abstract

Coastal ecosystems play significant ecological and economic roles but are threatened and facing decline. Microbes drive various biogeochemical processes in coastal ecosystems. Tidal flats are critical components of coastal ecosystems; however, the structure and function of microbial communities in tidal flats are poorly understood. Here we investigated the seasonal variations of bacterial communities along a tidal flat series (subtidal, intertidal and supratidal flats) and the factors affecting the variations. Bacterial community composition and diversity were analyzed over four seasons by 16S rRNA genes using the Ion Torrent PGM platform. Bacterial community composition differed significantly along the tidal flat series. Bacterial phylogenetic diversity increased while phylogenetic turnover decreased from subtidal to supratidal flats. Moreover, the bacterial community structure differed seasonally. Canonical correspondence analysis identified salinity as a major environmental factor structuring the microbial community in the sediment along the successional series. Meanwhile, temperature and nitrite concentration were major drivers of seasonal microbial changes. Despite major compositional shifts, nitrogen, methane and energy metabolisms predicted by PICRUSt were inhibited in the winter. Taken together, this study indicates that bacterial community structure changed along the successional tidal flat series and provides new insights on the characteristics of bacterial communities in coastal ecosystems.

Estuarine and coastal ecosystems in the world play vital ecological and economic roles. The global decline in coastal wetlands is affecting a number of critical benefits and ecosystem services provided by coastal ecosystems. Tidal flats are tidal dominant coastal wetlands and are characterized by high primary productivity and biological diversity in the sediment^1^. Tidal flats are sandwiched between marine, freshwater and land environments and are found in areas with low slopes and regular flooding^1^,^2^. Tidal flats are affected by habitat destruction, hydrological alteration, climate change, overexploitation, and pollution^3^.

Tidal flats are generally found several hundred meters wide along the coast, often forming a successional series consisting of a subtidal flat, an intertidal flat, and a supratidal flat in a landward direction^4^. The subtidal flat is below water in low tide and is seldom exposed subaerially and is un-vegetated^5^,^6^. The supratidal flat is above water in high tide and is affected intermittently by tidal action and vegetated with halophytes. The intertidal flat lies between the subtidal and supratidal flats and is submerged twice a day and is sparsely vegetated. Driven by tidal actions, tidal flats have natural geographical and environmental gradients along subtidal to supratidal flats, including changes in salinity, nutrient content, particle size distribution, duration of exposure of the sediment to the air, and vegetation type and coverage, among others^5^,^6^,^7^. In temperate tidal wetlands, there are large seasonal variations in environmental conditions, especially temperature^8^. Moreover, the supply of nutrients may also have large temporal variations due to seasonal processes, such as the allocation of photosynthesis into the sediment by plant roots and the seasonal input of organic matter from the sea^7^,^9^.

Microbes play critical roles in regulating ecological processes in tidal flats^5^. They support the main biogeochemical processes such as primary production and remineralization of organic matter, provide protection to larger organisms through the formation of biofilms, and influence the settlement of faunal larvae in tidal flats^10^,^11^,^12^,^13^. A large number of microorganisms in tidal flats contribute to the diets of invertebrates, fish, and shorebirds that live in coastal ecosystems^14^,^15^,^16^. Autotrophic bacteria, such as cyanobacteria, are abundant in tidal flats and have high rates of primary production, contributing to rapid carbon sequestration in tidal flats^17^,^18^. Microbial communities in tidal flats are widespread and complex, having great effects on coastal ecosystems^16^,^19^,^20^. However, there are knowledge gaps in understanding the structure and the ecological role of microbial communities in tidal flats due to large spatial and seasonal variations in those ecosystems.

We hypothesize that bacterial community structure and function have spatial and seasonal changes in the successional series of tidal flats from subtidal to intertidal to supratidal flat, reflecting systematic changes in site conditions along the successional series. In this study, we undertook an intensive field sampling in a successional series of tidal flats in the Yellow River Delta, which was affected by hydrodynamic forces of tides and considerable seasonal changes in temperature and other ecological factors. Here, we determined bacterial community in tidal flats by sequencing 16S rRNA gene amplicons with the Ion Torrent PMG platform and functional profiles predicted with PICRUSt. We analyzed these data to answer the following questions: (1) what are the spatial and seasonal changes of the bacterial community in a successional series of tidal flats? and (2) what are the dominant driving factors for those changes in tidal flats?

## Results

### Bacterial community composition in tidal flats

After quality filtering, a total of 266,468 reads were obtained from the 36 sediment samples, with an average sequence number of 7401 ± 1270 per sample. At 97% sequence identity, 7428 OTUs (Operational taxonomic units) were identified (Supplementary Fig. 2). Most samples were found to almost reach the saturated stage, suggesting that we sequenced almost all bacterial species in the samples. Four samples that were not saturated and thus with a small read numbers were removed from all analysis.

**Figure 1:**
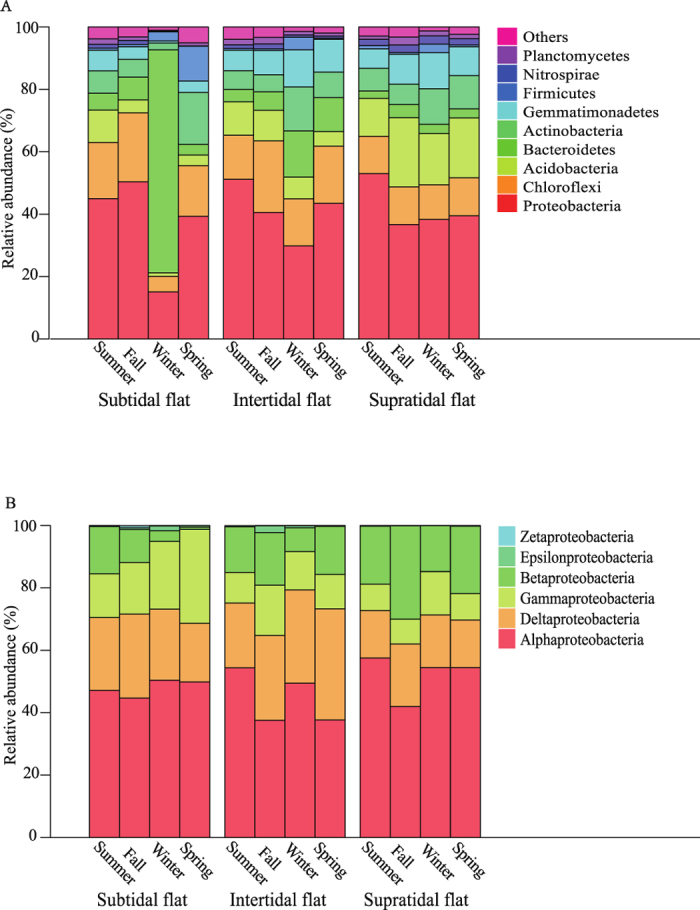
The relative abundances of (**A**) bacterial phyla and (**B**) Proteobacterial subphyla in different seasons in the successional tidal flat series.

The bacterial communities were dominated by phyla Proteobacteria (43.7%) and Chloroflexi (16.2%), followed by Bacteroidetes (11.8%), Acidobacteria (11.0%), and Actinobacteria (9.2%) (Fig. 1A). For the most abundant phylum Proteobacteria, we considered the relative abundance distributions at the subphyla level (Fig. 1B). Alphaproteobacteria dominated more approximate 50% of the Proteobacteria in the tidal flats. The relative abundances of Betaproteobacteria, Acidobacteria, Gemmatimonadetes, and Nitrospirae increased progressively from the subtidal to the supratidal flat (*P* < 0.001), while the relative abundances of Deltaproteobacteria, Gammaproteobacteria, and Bacteroidetes steadily decreased (*P* = 0.084). There were significant differences among seasons in Proteobacteria (*P* < 0.001), Chloroflexi (*P* = 0.012), Actinobacteria (*P* = 0.014), and Alphaproteobacteria (*P* = 0.030).

In winter, Bacteroidetes (71.5%) was the dominant bacterial phylum, followed by Proteobacteria (15.1%) and Chloroflexi (5.0%) in the subtidal flat. Comparing with other seasons, the relative abundance of Alphaproteobacteria, Actinobacteria, and Anaerolineae decreased, while the order Flavobacteriales increased in the subtidal flat in winter (Supplementary Fig. 3).

### Bacterial phylogenetic diversity in tidal flats

**Figure 2:**
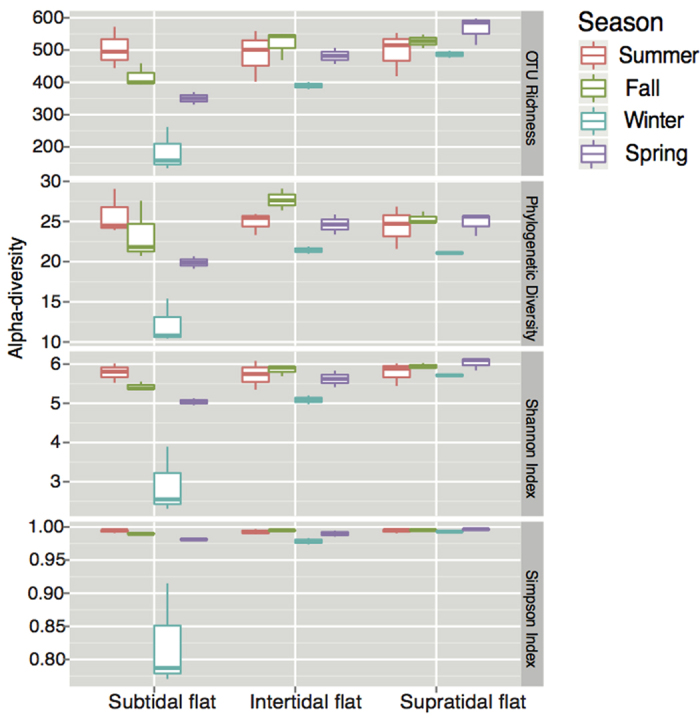
Box plots for alpha-diversity of the bacterial communities in different seasons in the successional tidal flat series. The ends of the whiskers represent the minimum and maximum, the bottom and top of the box are the first and third quartiles, and the line inside the box is the median.

Alpha-diversity of bacterial communities, such as OTU richness, Faith’s phylogenetic diversity (PD), Shannon’s index and Simpson index, was significantly lower in winter than in other sampling seasons in all tidal flat types, especially in subtidal flat (Fig. 2). The alpha-diversity was similar among different tidal flats in summer, and mostly decreased from the supratidal flat to the subtidal flat in other seasons.

**Figure 3:**
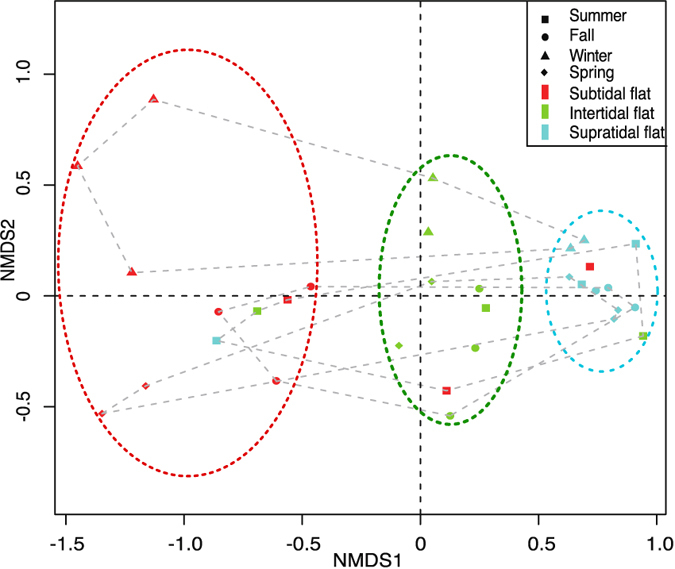
The NMDS plots for bacterial community structure based on Unifrac distance. Samples from three plots in the same tidal flat are represented by different colors. Samples from the four seasons are represented by different shapes of symbols.

To assess the bacterial phylogenetic beta-diversity, the NMDS analysis based on Unifrac distance was employed to study the phylogenetic similarities among the bacterial communities (Fig. 3). Bacterial communities were dissimilar between winter and other seasons (ANOSIM, *R* = 0.24, *P* = 0.001). Moreover, bacterial community in the same sampling plot was clustered together (ANOSIM, *R* = 0.17, *P* = 0.002), and the distances between bacterial communities represented tidal flat locations. The ANOSIM results suggested that the differences between seasons were greater than that between tidal flats, indicating that the bacterial community was more sensitive to seasonal variation in temperature. In addition, the temporal variation in phylogenetic dissimilarities (phylogenetic turnover) were the highest in the subtidal flat as compared with other tidal flats, as the dots across triplicates and sampling times in the subtidal flat were the least clustered than those observed in other tidal flats.

### Contribution of environmental factors to bacterial community structure

Our study indicated that environmental factors might be important determinants of bacterial community structure in the coastal wetland. We sought to identify environmental factors contributing to the variations of bacterial community structure in tidal flats by quantifying environmental variables and performing a canonical correspondence analysis (CCA)^12^.

**Figure 4:**
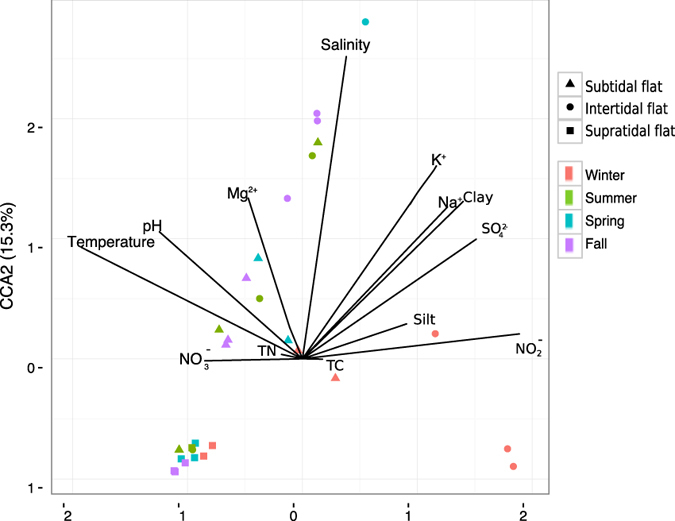
Canonical correspondence analysis (CCA) performed on the sediments in the successional tidal flat series using bacterial community structure and environmental factors.

The CCA explained 38.2% of the variation in the first two axes (Fig. 4) and confirmed the clear separation of sites according to environmental factors. Axis 1 (CCA1) established a separation between samples collected in winter and other seasons, indicating that temperature and nitrite concentration may act as the most important drivers of seasonal changes of the microbial community. Axis 2 (CCA2) separated samples along the successional series of tidal flats. Salinity was identified as a major environmental factor structuring the microbial community in the sediment along the successional series.

### Imputed functional profiles in tidal flats

**Figure 5:**
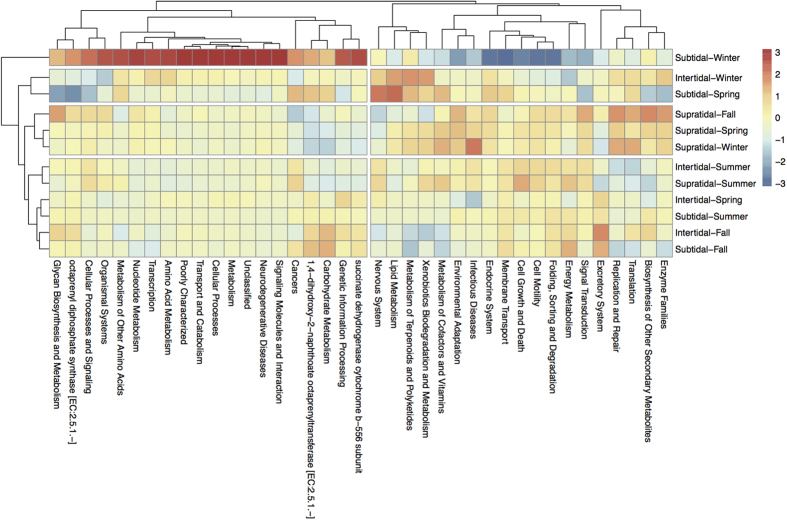
The heat-map of normalized relative abundance of imputed functional profiles using PICRUSt grouped into level-3 functional categories.

**Figure 6:**
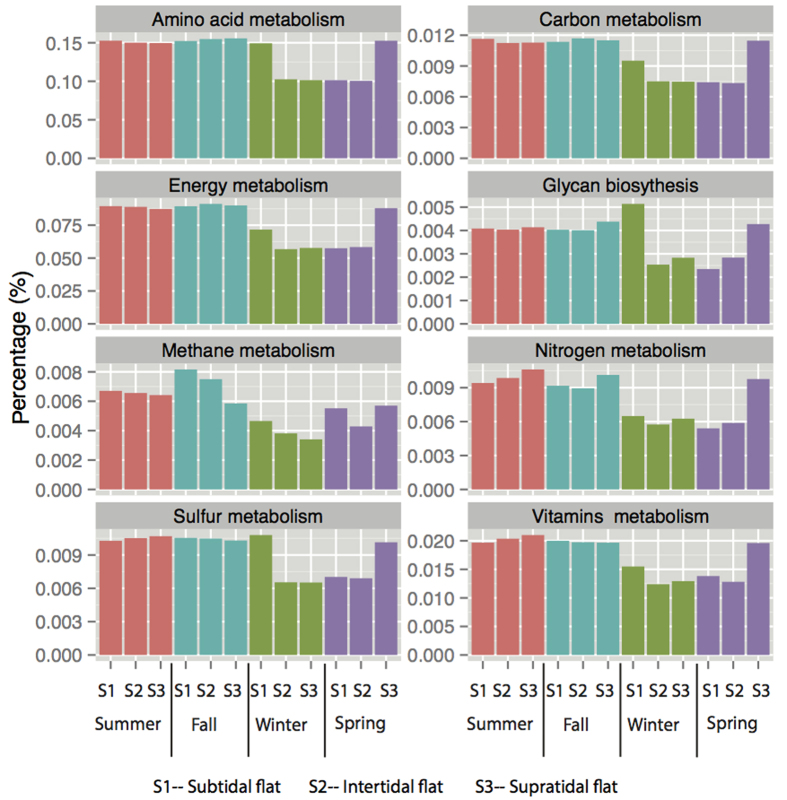
The relative abundance of some imputed functional profiles in different seasons in the successional tidal flat series.

Based on the 16S rRNA gene copy number of detected phylotype, we predicted the functional profiles of bacterial communities in tidal flats. The relative abundances of functional profiles were similar in most of samples. The functional profiles were different between in subtidal flat in winter and in other samples (Fig. 5). They were clustered into two groups in which one was overrepresented and the other was underrepresented in subtidal flat in winter. The overrepresented group in subtidal flat in winter included transcription, signaling molecules and interaction, transport and catabolism, and some metabolisms (nucleotide metabolism, amino acid metabolism, glycan biosynthesis and metabolism). The underrepresented group included membrane transport and signal transduction belonging to environmental information processing, cell motility and growth and death belonging to cellular processes, environmental adaptation and metabolisms such as energy metabolism, lipid metabolism, metabolism of cofactors and vitamins, xenobiotics biodegradation and metabolism. We also analyzed functional profiles that were involved in the bacterial community adaptation to environment and nutritional conditions (Fig. 6). The relative abundance of vitamin metabolism, energy metabolism, nitrogen metabolism, and methane metabolism varied among seasons (*P* < 0.05), and were the lowest in winter.

## Discussion

A reduction in bacterial diversity and a consistent shift of community structure from the supratidal flat to the subtidal flat resulted from a combination of factors associated with coastal environment, especially tidal action. The rising tidal water causes numerous environmental stresses to bacterial community, such as high salinity, pH and ionic strength^5^. On the other hand, tidal action could increase the daily temperature difference and cause alternating aerobic and anaerobic conditions^21^. Under anaerobic conditions, nutrient bioavailability and soil enzyme activities decrease^7^. In addition, seawater brought in by tides brings specific ions into the tidal flats, which may affect microbial functional groups^7^,^19^,^22^. For example, the growth of sulfate-reducing bacteria promoted by increasing sulfate from seawater could outcompete the methanogens typically found in freshwater wetlands^12^,^21^. Noticeably, the high complexity of the bacterial communities in some marine coastal environments has been explained by the intrinsically high and stochastic microbial influx from the sea, when bacterial activity is at a low level^10^. In addition, the observed patterns in bacteria community structure in the subtidal flat might also have been caused by the partial colonization by microbial mats^7^,^17^. The occurrence and distribution of microbial mats may be related to input of marine organisms such as microbial influx, algae growth, and movement of fiddler crabs throughout the year^23^,^24^.

The lowest bacterial phylogenetic diversity in winter in tidal flats is similar to the findings on bacterial communities in coastal water^25^ and lake sediments^26^. The decrease of bacterial phylogenetic diversity in low temperature was likely because bacteria are in an inferior position in competition with fungi when the temperature is low^27^. Moreover, rare bacterial taxa are difficult to be detected with limited sequencing depth by virtue of the low biomass of bacteria in low temperature^5^,^26^.

The increase of bacterial phylogenetic turnover (seasonal variation of bacterial communities) from supratidal flat to subtidal flat was likely due to the different degree of influence of tidal actions. The high variations in the subtidal flat were associated with tidal flooding twice a day, which causes temporal variability in nutrients coming from marine organisms and invasion by diverse marine bacteria^28^. Furthermore, the high variations in the subtidal flat could also be explained by higher amplitude of variation in environmental conditions in poorly vegetated sites. The heterogeneous erodibility of the surficial sediment can change the sediment microtopography and make the sediment to be more frequently disturbed, and that is one possible reason for the higher phylogenetic turnover rate in the subtidal flat^29^. With the buffering effects of the sediment and as plants become more abundant in the intertidal and supratidal flats, the magnitude of variation reduced, resulting in low phylogenetic turnover. Loss of diversity and increase of turnover rate will make the microbial community fragile and the ecosystems vulnerable to environmental change, and increase the risk of abrupt and potentially irreversible ecosystem collapse^5^,^30^.

Temperature and nitrite were recognized as major environmental factors influencing the seasonal changes of bacterial community in tidal flats in the Yellow River Delta. Temperature has long been regarded as an important factor driving changes in bacterial community structure and function^31^. Seasonal changes in temperature can be directly reflected in bacterial community diversity and function^5^. For example, microbial community diversity and structure in lake sediments varied between winter and summer in Lake Erie^26^. Nitrite is involved in nitrification and de-nitrification processes, which are the primary transformation route in wetlands, especially in wetlands with alternating anaerobic and aerobic conditions^32^,^33^. Actinobacteria and Gemmatimonadetes were positively correlated with nitrite in freshwater wetlands^24^.

Pervious studies reported that salinity is the one of the dominant factors controlling the microbial community structure^34^, especially in coastal regions^35^,^36^. Salinity can change physicochemical properties and microbial community composition in the sediment. High salinity can directly suppress the heterotrophic metabolic capabilities^34^,^37^, and decrease the diversity of heterotrophic bacteria species, such as Beta-proteobacteria, Chloroflexi, and Bacteroidetes^38^. Our results are consistent with earlier findings that salinity could affect the spatial changes of bacterial community structure in the tidal flats in the Yellow River Delta

The difference of phylogenetic groups in various tidal flats might reflect the functional discrepancy of bacterial communities along the successional series of tidal flats. The high abundance of Deltaproteobacteria and Gammaproteobacteria suggests that chemoautotrophic bacteria flourish in the subtidal flat, which have abundant anaerobic sulfur-reducing bacteria^11^,^33^. In addition, the Flavobacteriales in Bacteroidetes have been shown to have high abundances in subtidal flats in winter, which are very well known to be a result of a large number of cold-adapted genera in the marine ecosystems^39^,^40^,^41^.

The pattern of bacteria community in the subtidal flat is also consistent with recent findings in sediments in mangrove systems^12^,^21^ and salt marshes^10^,^12^,^13^. Comparing to the subtidal flat, the more abundant phyla in the supratidal flat, such as Betaproteobacteria, Acidobacteria, Gemmatimonadetes and Nitrospirae, have been reported to be prevalent in the sediment environment^19^,^42^. Alphaproteobacteria and Betaproteobacteria are generally considered as indicators of soils with high nutrients and low pH^2^. Acidobacteria is ubiquitous and abundant members in soil bacterial communities with a few described strains^33^. Gemmatimonadetes has been reported to positively correlate with nitrite concentration in anaerobic environments^24^. Nitrospirae, a group of nitrite oxidizing bacteria, is one of the key players in the nitrogen cycle and quite abundant in freshwater sediments^12^,^43^. The intermediate abundance of all communities in intertidal flats indicates the developing role of the intertidal flat in the coastal ecosystem.

Compared with other studies, the relative abundance of Cyanobacteria was rather low, representing only 0.0034% of the OTU. This result is similar to a pervious study in the Yellow River Delta^38^. It could be explained by the low nutrient content in the Yellow River Delta that inhibits the growth of Cyanobacteria^18^,^39^. The loss of Cyanobacteria in the sediment may also be due to the large amount of fine particles brought in by the Yellow River. These fine particles (clay and silt) reduce the amount of light available to the cyanobacteria, reducing their capacity for photosynthesis and therefore reducing their abundance^17^.

PICRUSt analysis predict high relative abundances of imputed functional profiles in the subtidal flat related to cellular processes, transport and catabolism, and nucleotide and amino acid metabolism, and the decrease of those related to metabolisms of nitrogen, methane, vitamin and energy. Our results showed the bacterial community responses to low temperature in tidal flats, especially in the subtidal flat. Similar observations have been reported for marine and lake ecosystems^12^,^15^,^38^. These functional profiles in subtidal flat in winter were generally found at low temperature, high ionic concentration, and poor nutrient conditions^31^,^42^,^44^, indicating that the bacterial community function in the subtidal flat was impaired in winter. The enzymes and microbial community in sediments have a lower activity at low temperatures, lowering the microorganism metabolism in winter^5^,^10^,^11^,^36^. Low temperature can be an environmental stress that affects microbial community structure and activity^45^. In low temperature, nutrient bioavailability is limited and microbial function in biogeochemical processes is influenced, leading to the reduction of the abundances of genes for related metabolisms^17^,^46^. It is important to keep in mind that these shifts of bacterial function could be due to low temperature and tidal actions that may structure the bacterial communities^5^,^7^,^10^. Bacteroidetes, tolerant to low temperature and posed high competition capacity in aquatic ecosystems^47^, made up more than 70% of the abundance of the bacterial community in our study. In winter, the reduced nutrient taken by tidal water could influence the competition between microbes^1^,^6^, and affect the microbial community structure. At the ecosystem level, these effects could alter microbial energy requirements, thus influencing nutrient availability and nutrient cycling if energy is shunted towards cellular processes and away from growth in the tidal flats. Additionally, microbes are at the bottom of the food chain; the changes of their diversity would influence the rest of the food chain in the tidal flats. The different microbial diversities are the result of many biological traits to adapt, survive, and replenish under the predation pressure, inter- and intraspecific competition caused by the temporal and spatial environmental changes in the tidal flats.

However, the majority of predicted functional profiles were redundant between bacterial communities spanning the successional series of tidal flats. A growing body of evidence shows that the stable functional profile is due to the existence of functional gene redundancy among bacterial communities^16^,^48^,^49^. This may indicate that bacterial communities include a reservoir of species with the potential to restore or repair perturbed ecological processes, which may arise with environmental change^22^,^50^. In addition, microbial community function has physiological plasticity to quickly respond to environmental changes and has the potential to acquire new genes through recombination^9^,^10^,^19^. That is, stress resilience of microbial functional profiles supported by greater phylogenetic diversity^51^. PICRUSt cannot replace whole metagenome profiling and could be problematic when analyzing microbial communities with a large proportion of poorly characterized members. However, it is useful to supplement 16S rRNA analyses in metagenome studies, especially for broad surveys in microbial ecology applications.

In conclusion, bacterial community structure significantly shifted along the successional series of tidal flats and among the different seasons, with the bacterial phylogenetic diversity sharply decreased in winter in the subtidal flat comparing with other seasons or the other two types of tidal flats. The CCA indicated that salinity had a considerable impact on the microbial community in the sediment along the successional series. Moreover, temperature and nitrite concentration were primary drivers of seasonal microbial changes in the studied tidal flats. Our study demonstrated that tidal activity altered the structure and function of bacterial community in tidal flats. These observations thus provide new insights about the microbial community-environmental condition relationships in tidal flats, unique ecosystems in the coastal region and offer predicting case study for understanding the impact of environmental changes on bacterial community structure and function in coastal ecosystems.

## Materials and Methods

### Study area

The Yellow River Delta, located in northeast Shandong Province of China, has the largest and youngest coastal wetlands in the country, with some of the large river deltas in the world. The Yellow River Delta has a warm-temperate and semi-humid continental monsoon climate, with annual average temperature of 11.7–12.6 °C. The average temperatures in the four seasons are 27.4 (summer), 11.7 (fall), −0.3 °C (winter) and 20.1 °C (spring). The annual potential evaporation is 1900–2400 mm and annual precipitation is 530–630 mm, of which 70% is rainfall that occurs between June and August. Tidal flats are the dominant wetland type, covering more than 60% of this delta^52^.

### Sampling and physicochemical analyses

This study was carried out in the northern Yellow River Delta (118.6^o^ E-119.3^o^ E, 37.6^o^ N-38.2^o^ N). Sediment samples (n = 36) were collected in July 2012 (summer), October 2012 (fall), and February 2013 (winter), May 2013 (spring) in a typical tidal flat located in the northern Yellow River Delta (118.6^o^ E-119.3^o^ E, 37.6^o^ N-38.2^o^ N), China. The study site presents a well-characterized tidal flat in recent years^53^.

We established triplicate plots (5 × 5m^2^) at each successional tidal flat series: subtidal flat, intertidal flat, and supratidal flat (separated 25 m from each other). Location and detailed information of each tidal flat are provided in Table 1 and Supplementary Fig. 1. In each plot, sediment samples were collected by randomly taking 5 soil cores using a sterile sampling device. Considering the vegetation effect, soil cores were collected from 0–20 cm depth. Collected soil cores per plot were pooled in a sterile plastic bag, sealed and transported to the laboratory within 24 h. Soil core was homogenized and plant debris were removed by hand. Homogenized samples per plot (n = 36) were stored at −80 °C for total DNA extraction within 30 days and at −4 °C for ammonium, nitrite, and nitrate analysis with in 7 days. A subset of the soil was air-dried and sieved (2 mm mesh size) for physicochemical analysis. To avoid human factors to the bacterial community composition, the subset of sediment for DNA extraction was manually homogenized but not sieved. In this study, no spatial variations within plots were considered. Variations within successional stages were addressed through triplicated plots (spatial) and across sampling times (temporal).

Soil water content (%) was determined by oven drying at 105 °C. Salinity of sediment was quantified as electrical conductivity (EC, μS cm^−1^) with a 1:5 soil-to-water ratio, and pH was measured using a glass electrode. Particle size distribution was determined by the laser diffraction method. Inorganic ions (Ca^2+^, Mg^2+^, K^+^, Na^+^, Cl^−^, and SO_4_^2−^) were extracted and analyzed by ion chromatography. Soil ammonium (NH_4_^+^), nitrate (NO_3_^−^) and nitrite (NO_2_^−^) were extracted from fresh soils with 2 M KCl and determined on an Astoria Analyzer 300 system. Sediment samples were air-dried and sieved to 2 mm for determination of total carbon (TC) and total nitrogen (TN) on an elemental analyzer (Vario El, Elementar Co., Germany), and organic matter (OM) by K_2_CrO_7_ oxidation-colorimetric method. All measurements were replicated three times for each sample.

### 16S rRNA gene and bacterial community analysis

Microbial genomic DNA was extracted, isolated with the FastDNA SPIN kit for soil (MP Biomedicals, Solon, OH, USA). Sample processing followed the manufacturer’s instructions. After isolation, the purified DNA was eluted in 100 μL of elution buffer. Quality and purity of the isolated genomic DNA was confirmed by agarose gel-electrophoresis and spectrophotometry on a NanoDrop 2000 device (Fisher Scientific, Schwerte, Germany).

The V3 region of the Bacterial 16S rRNA gene was amplified by polymerase chain reaction (PCR) using the V3 region specific oligonucleotide primers. We amplified a standard 180 bp V3 region using primers 338F and 518R and amplification conditions^54^,^55^. The forward primers 338F were modified by the addition of a PGM sequencing adaptor, a ‘GT’ spacer and a unique error correcting Golay barcode to allow multiplex analyses^56^. Prior to sequencing, all amplicons were assessed for fragment size distribution and DNA concentration using a Bioanalyser 2100 (Agilent Technologies, USA). The samples were adjusted to a final concentration of 10 pM and were attached to the surface of Ion Sphere Particles (ISPs) using an Ion Xpress Template 100 kit (Life Technologies, USA) according to the manufacturer’s instructions. Manual enrichment of the resulting ISPs resulted in >95% templated-ISPs. Templated-ISPs were sequenced on a “316” (100 Mbp) micro-chip using the Ion Torrent Personal Genome Machine (Life Technologies, USA) for 65 cycles (260 flows). After sequencing, the individual sequence reads were filtered within the PGM software to remove low quality and polyclonal sequences. Sequences matching the PGM 3′ adaptor were also automatically trimmed. All PGM quality filtered data were exported as FastQ files, split into constituent *.fasta and *.qual files and subsequently analyzed using the QIIME pipeline^55^,^57^. Quality filtering of the reads was performed at Q25, before the grouping into operational taxonomic units (OTUs) at a 97% sequence homology cut-off.

### Imputation of microbial functional profiles

This study employed PICRUSt (Phylogenetic Investigation of Communities by Reconstruction of Unobserved States) to predict functional differences in the tidal flat gradient^58^. The OTU table was used as the input file for metagenome imputation of individual sediment samples. Predicted gene class abundances were analyzed at KEGG (Kyoto Encyclopedia of Genes and Genomes) Orthology group level 3. The mean nearest sequenced taxon index was lower (0.14 ± 0.04) than that reported for sediment bacterial communities (0.17 ± 0.02)^55^.

### Statistical analyses

All statistical analyses and graphics were done using the R program (http://www.r-project.org). Depth of sequencing coverage was quantified by rarefaction curve using the vegan package. The taxonomic diversities of bacterial communities were calculated with the *vegan* package. The phylogenetic diversities of communities were calculated using the *picante* package. Non-metric multidimensional scaling (NMDS) analyses was used for ordination based on the Unifrac phylogenetic distance matrix for bacterial community structure. Analysis of similarity (ANOSIM) was employed for group difference test between community groups. To determine factors controlling bacterial communities in tidal flats, we employed canonical correspondence analysis (CCA) in the *vegan* R package and kept the variables that are mostly independent from the other environmental variables entering in the model.

## Additional Information

**Accession codes:** These sequence data have been submitted to the SRA databases under accession number SRX1058187.

**How to cite this article**: Lv, X. *et al*. Bacterial community structure and function shift along a successional series of tidal flats in the Yellow River Delta. *Sci. Rep.* **6**, 36550; doi: 10.1038/srep36550 (2016).

**Publisher’s note:** Springer Nature remains neutral with regard to jurisdictional claims in published maps and institutional affiliations.

## Supplementary Material

Supplementary Information

## Figures and Tables

**Table 1:** General condition of the sampling sites along a successional tidal flat series.

	Subtidal flat	Intertidal flat	Supratidal flat
Site code	S1	S2	S3
Dominant plant species	None	*Tamarix chinensis*	*Phragmites australis*
Vegetation cover (%)	0	35	100
Elevation (m)	2.2	2.6	3.2
Distance to the subtidal line (m)	0	200	670
