# Supplementary material for: Bacterial community structure and function shift along a successional series of tidal flats in the Yellow River Delta
Source: Sci Rep. 2016 Nov 8;6:36550. doi: 10.1038/srep36550 (PMC5099912; doi:10.1038/srep36550)
Supplement: Supplementary Information [file srep36550-s1.pdf]

Supplementary Material

Bacterial community structure and function shift along a successional series of tidal flats in the Yellow River Delta

- Xiaofei Lyu[1, 2, 3], Bin Ma[1,3], Junbao Yu[4*], Scott X. Chang[3], Jianming Xu[1], Yunzhao Li[4], Guangmei Wang[2], Guangxuan Han[2], Guan Bo[2], Xiaojing Chu[2]
- [1] College of Environmental and Resource Sciences, Zhejiang Provincial Key Laboratory of Agricultural Resources and Environment, Zhejiang University, Hangzhou, Zhejiang, 310012, China
- [2] Key Laboratory of Coastal Environmental Processes and Ecological Remediation, Yantai Institute of Coastal Research Chinese Academy of Sciences, Yantai, Shandong, 264003, China
- [3] Department of Renewable Resources, University of Alberta, Edmonton, Alberta, T6G 2E3, Canada
- [4] School of Geography and Planning, Ludong University, Yantai, Shandong, 264001, China

- **Correspondence:** Junbao Yu: junbao.yu@gmail.com

Supplementary Figures

Legends for Supplementary Figure

Supplementary Figure 1. The locations of the sampling sites, represented by the solid dots.

Supplementary Figure 2. Rarefaction curves for soil bacterial communities in tidal flats using 16S rRNA gene sequences at 3% difference levels.

Supplementary Figure 3. The seasonal changes of the relative abundance of components in bacterial communities in subtidal flat at domian (red) to Phylum (blue), Class (yellow), and order (green) taxonomic levels.

Supplementary Figure 4. The soil properties in different seasons in tidal flats. The ends of the whiskers represent the minimum and maximum of all of the data. The bottom and top of the box are the first and third quartiles, and the band inside the box is the median.

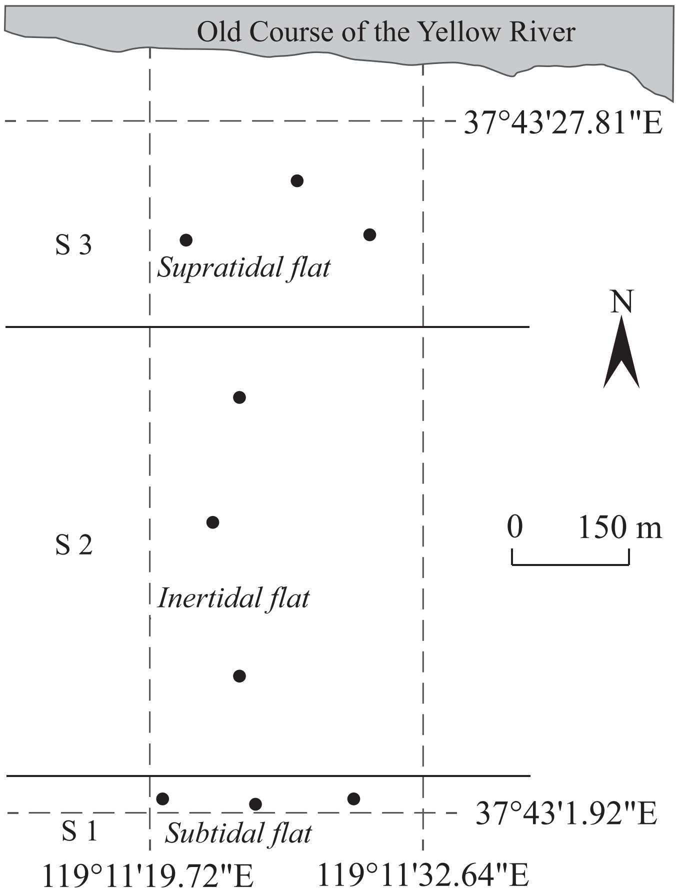

Supplementary Figure 1. The locations of the sampling sites, represented by the solid dots.

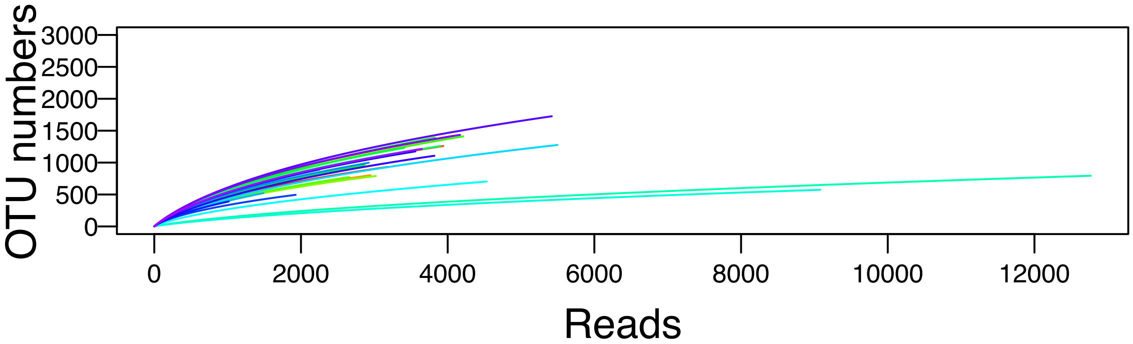

Supplementary Figure 2. Rarefaction curves for soil bacterial communities in tidal flats using 16S rRNA gene sequences at 3% difference levels.

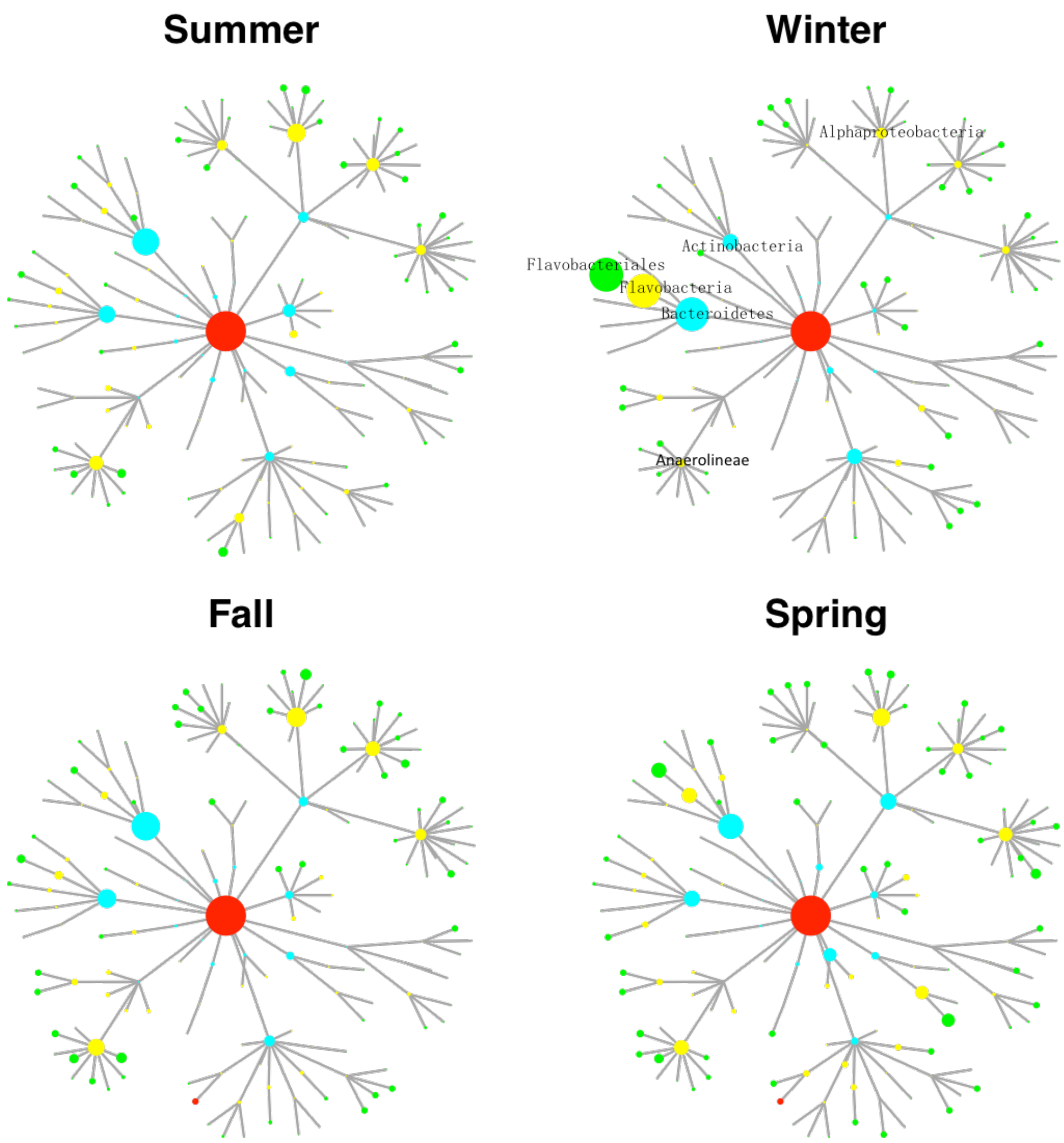

Supplementary Figure 3. The seasonal changes of the relative abundance of components in bacterial communities in subtidal flat at domian (red) to Phylum (blue), Class (yellow), and order (green) taxonomic levels.

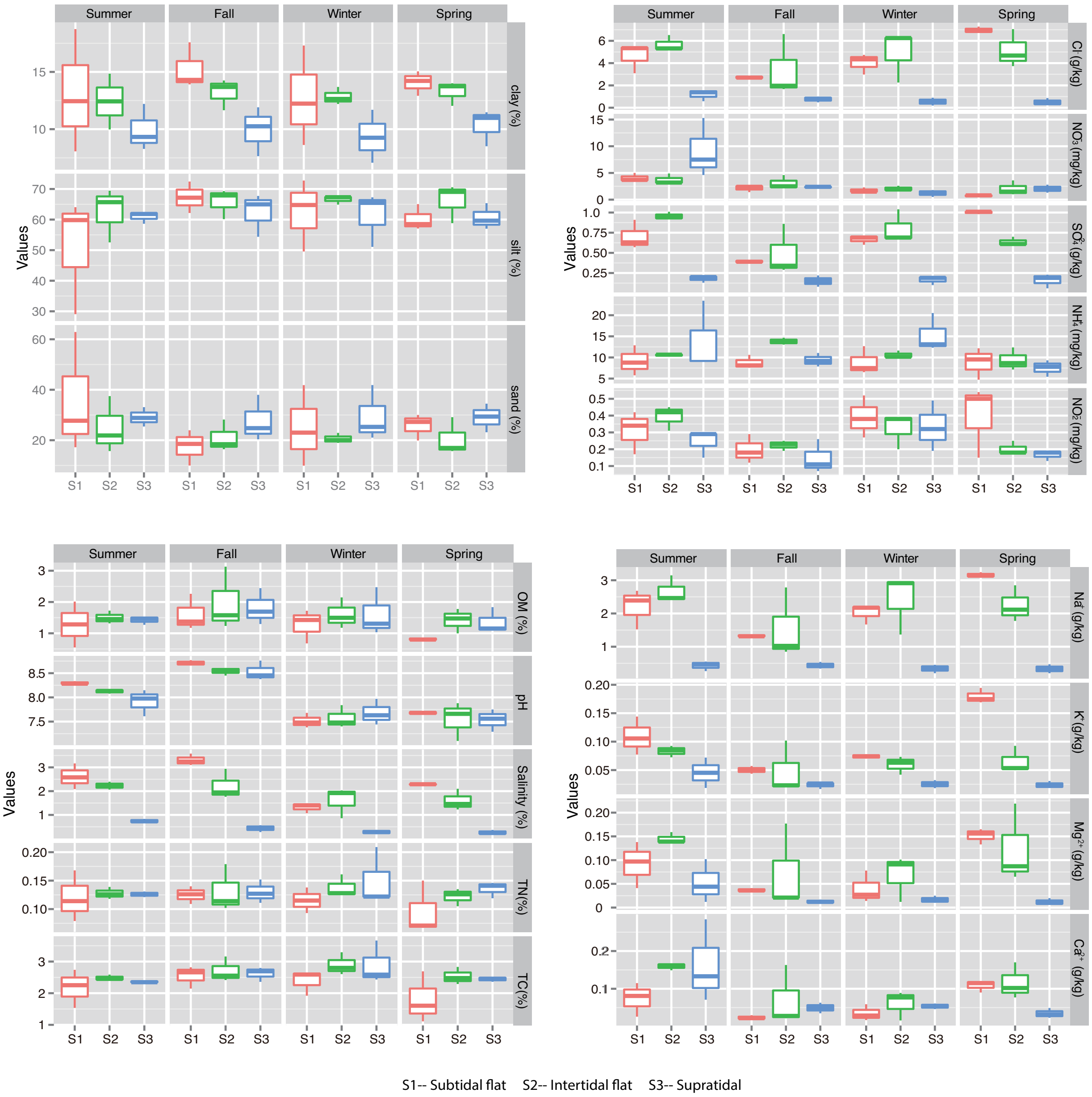

Supplementary Figure 4. The soil properties in different seasons in tidal flats. The ends of the whiskers represent the minimum and maximum of all of the data. The bottom and top of the box are the first and third quartiles, and the band inside the box is the median.
